# Supplementary material for: Use of Online Food Delivery Services to Order Food Prepared Away-From-Home and Associated Sociodemographic Characteristics: A Cross-Sectional, Multi-Country Analysis
Source: Int J Environ Res Public Health. 2020 Jul 17;17(14):5190. doi: 10.3390/ijerph17145190 (PMC7400536; doi:10.3390/ijerph17145190)
Supplement: Supplementary file 1 [file ijerph-17-05190-s001.pdf]

Table S1: Sociodemographic characteristics of sample (n=19,378). Data are from the International Food Policy Study, collected in 2018 [a].

	Australia (n=3578)		Canada (n=3698)		Mexico (n=3515)		UK (n=4694)		USA (n=3893)		Total (n=19378)	
Sex												
Male	1732	(48.4)	1835	(49.6)	1690	(48.1)	2249	(47.9)	1860	(47.8)	9367	(48.3)
Female	1847	(51.6)	1863	(50.4)	1824	(51.9)	2444	(52.1)	2032	(52.2)	10010	(51.7)
Ethnicity												
Majority	2752	(76.9)	2972	(80.4)	2828	(80.5)	4211	(89.7)	2975	(76.4)	15738	(81.2)
Minority	826	(23.1)	726	(19.6)	687	(19.5)	483	(10.3)	918	(23.6)	3640	(18.8)
Age (years)												
Median (IQR)	48 (33-61)		51 (34-62)		38 (28-50)		50 (34-64)		49 (32-62)		47 (32-61)	
Education												
Low	1512	(42.3)	1514	(40.9)	666	(18.9)	2269	(48.3)	2261	(58.1)	8222	(42.4)
Medium	1171	(32.7)	1256	(34.0)	423	(13.2)	1091	(23.2)	390	(10.0)	4370	(22.6)
High	895	(25.0)	928	(25.1)	2387	(67.9)	1333	(28.4)	1242	(31.9)	6785	(35.0)
BMI (kg/m^2)												
Not overweight (≤ 24.9)	1422	(39.7)	1383	(37.4)	1551	(44.1)	1802	(38.4)	1364	(35.0)	7522	(38.8)
Overweight (25.0-29.9)	990	(27.7)	1107	(29.9)	1099	(31.2)	1306	(27.8)	1160	(29.8)	5661	(29.2)
Obesity (≥ 30.0)	780	(21.8)	928	(25.1)	562	(16.0)	818	(17.4)	1092	(28.1)	4180	(21.6)
Missing	386	(10.8)	280	(7.6)	304	(8.7)	767	(16.3)	277	(7.1)	2015	(10.4)
Child <18 years in home												
No	2670	(74.6)	2931	(79.2)	1723	(49.0)	3564	(75.9)	2963	(76.1)	13850	(71.5)
Yes	909	(25.4)	767	(20.8)	1792	(51.0)	1130	(24.1)	930	(23.9)	5528	(28.5)

[a] Unless stated data reported as n (%).

Table S2: Sociodemographic characteristics of non-online food delivery service customers (n=12,163) amongst sample (n=19,378). Data are from the International Food Policy Study, collected in 2018 [a].

	Australia (n=3578)	Canada (n=3698)	Mexico (n=3515)	UK (n=4694)	USA (n=3893)	Total (n=19378)	p value for difference [b]
Non-online food delivery service customers	2188 (61.2)	2420 (65.4)	2396 (68.2)	2439 (52.0)	2721 (69.9)	12163 (62.8)	p>0.0001
Variable							
Sex							p>0.0001
Male	1011 (46.2)	1236 (51.1)	1174 (49.0)	1167 (47.9)	1278 (47.0)	5866 (48.2)	
Female	1177 (53.8)	1185 (48.9)	1222 (51.0)	1271 (52.1)	1443 (53.0)	6298 (51.8)	
Ethnicity							p>0.0001
Majority	1706 (77.9)	1930 (79.8)	1985 (82.8)	2167 (90.1)	2130 (78.3)	9947 (81.8)	
Minority	483 (22.1)	490 (20.2)	411 (17.2)	242 (9.9)	591 (21.7)	2216 (18.2)	
Age (years)							
Median (IQR)	48 (34-61)	48 (33-61)	40 (28-52)	50 (36-64)	50 (33-61)	47 (32-60)	
Education							p>0.0001
Low	915 (41.8)	953 (39.4)	508 (21.2)	1075 (44.1)	1591 (58.5)	5042 (41.5)	
Medium	725 (33.1)	824 (34.0)	326 (13.6)	591 (24.2)	285 (10.5)	2751 (22.6)	
High	548 (25.0)	644 (26.6)	1562 (65.2)	773 (31.7)	844 (31.0)	4370 (35.9)	
BMI (kg/m²)							p>0.0001
Not overweight (≤24.9)	855 (39.1)	884 (36.5)	1035 (43.2)	910 (37.3)	927 (34.1)	4611 (38.8)	
Overweight (25.0-29.9)	601 (27.5)	736 (30.4)	758 (31.6)	718 (29.4)	827 (30.4)	3460 (29.2)	
Obesity (≥30.0)	502 (23.0)	623 (25.7)	385 (16.1)	429 (17.6)	788 (29.0)	2727 (21.6)	
Missing	230 (10.5)	178 (7.3)	218 (9.1)	382 (15.7)	178 (6.5)	1185 (10.4)	
Child <18 years in home							
No	1646 (75.2)	1893 (78.2)	1302 (54.3)	1879 (77.0)	2108 (77.5)	8828 (72.6)	p>0.0001
Yes	542 (24.8)	528 (21.8)	1094 (45.7)	460 (23.0)	612 (22.5)	3335 (27.4)	

[a] Non-online food delivery service customers had purchased at least one meal prepared away-from-home directly from a food outlet, but not through an online food delivery service, in the past 7-days. Unless stated data reported as n (%).

[b] p values from Pearson's χ^2 test.

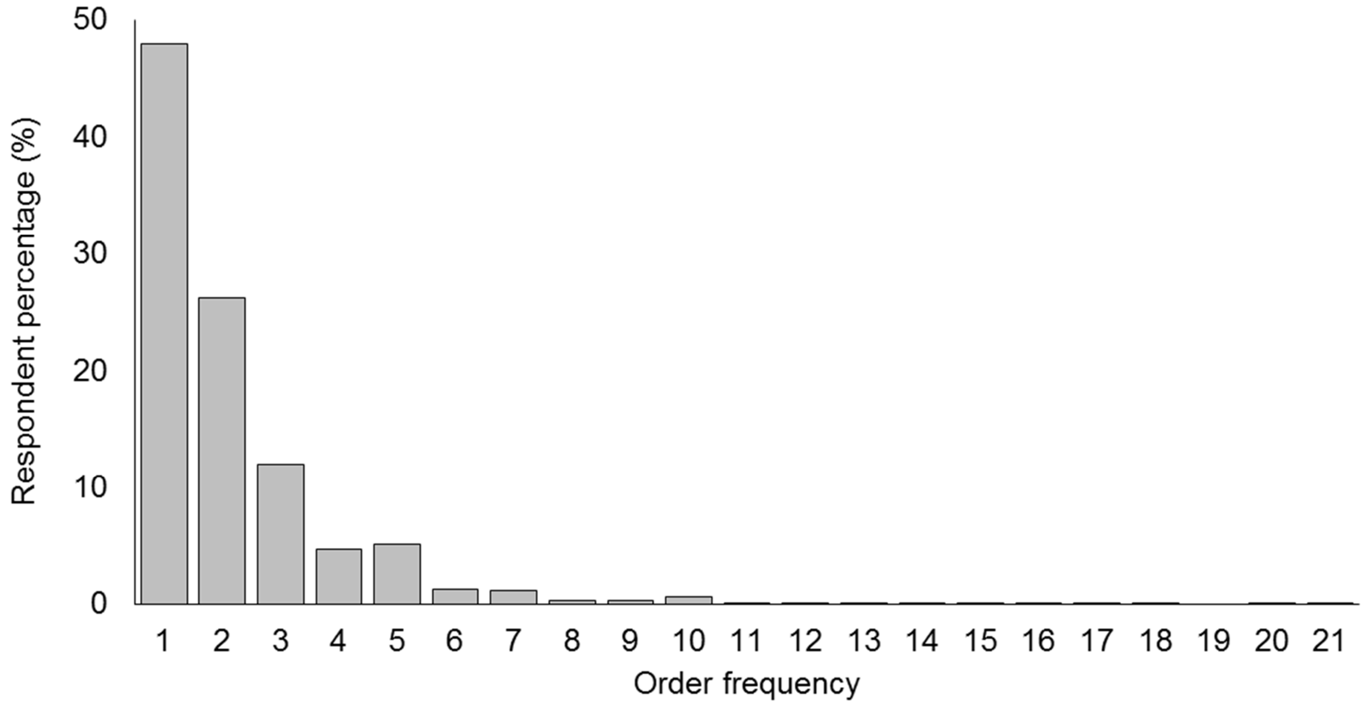

Figure S1: Frequency of online food delivery service use in the past 7-days amongst online food delivery service customers (n=2929). Data are from the International Food Policy Study, collected in 2018.

Table S3: Associations between prevalence of any online food delivery service use in the past 7-days and sociodemographic characteristics amongst sample (n=19,378). Data are from the International Food Policy Study, collected in 2018, analysed using logistic regression.

	Model 0 [a]		Model 1 [a]	
Variable and category	OR[b]	95% CI	OR[b]	95% CI
Sex (male)	1.41	1.28, 1.55	1.54	1.38, 1.71
Age (years)	0.94	0.94, 0.95	0.95	0.94, 0.95
Education				
Low	ref	-	-	-
Medium	1.23	1.06, 1.42	-	-
High	2.57	2.28, 2.90	-	-
Ethnicity (minority)	2.35	2.10, 2.63	1.64	1.45, 1.86
BMI (kg/m^2)				
Not overweight (≤ 24.9)	ref	-	ref	-
Overweight (25.0-29.9)	0.68	0.61, 0.76	0.90	0.79, 1.03
Obesity (≥ 30.0)	0.53	0.46, 0.61	0.87	0.75, 1.01
Missing	1.05	0.90, 1.23	1.01	0.85, 1.20
Child <18 in home (yes)	3.84	3.48, 4.23	2.85	2.57, 3.17
Country				
Australia	0.85	0.74, 0.99	0.74	0.61, 0.85
Canada	0.51	0.44, 0.60	0.46	0.39, 0.55
Mexico	1.80	1.58, 2.06	1.01	0.87, 1.18
UK	ref	-	ref	-
USA	0.71	0.61, 0.82	0.61	0.52, 0.72

[a] Model 0 = unadjusted. Model 1 = adjusted for all independent variables except education.

[b] = Odds Ratio

Table S4. Odds ratio (OR) and 95% confidence intervals (CIs) from two-way interaction term added to separate, maximally adjusted, logistic regression models. Data are from the International Food Policy Study, collected in 2018

Interaction term [a]	OR	95% CI
Sex x country (female = reference)		
Male x Australia	1.26	0.91, 1.76
Male x Canada	0.96	0.67, 1.37
Male x Mexico	0.66	0.50, 0.88
Male x USA	1.12	0.80, 1.55
Ethnicity x country (majority = reference)		
Minority x Australia	0.72	0.48, 1.08
Minority x Canada	0.90	0.60, 1.36
Minority x Mexico	0.92	0.62, 1.37
Minority x USA	1.13	0.77, 1.65
Age x country (continuous)		
Age x Australia	1.00	0.99, 1.02
Age x Canada	1.01	0.99, 1.02
Age x Mexico	1.03	1.02, 1.05
Age x USA	1.00	0.99, 1.02
Child <18 years at home x country (no = reference)		
Yes x Australia	0.82	0.59, 1.14
Yes x Canada	0.80	0.56, 1.14
Yes x Mexico	1.19	0.87, 1.61
Yes x USA	1.22	0.88, 1.69
Education x country (low = reference)		
Medium x Australia	1.47	0.94, 2.30
Medium x Canada	1.71	1.05, 2.77
Medium x Mexico	2.00	1.21, 3.31
Medium x USA	2.04	1.30, 3.20
High x Australia	2.10	1.40, 3.13
High x Canada	1.66	1.06, 2.60
High x Mexico	2.42	1.66, 3.55
High x USA	2.84	1.97, 4.08
BMI x country (not overweight = reference)		
Overweight x Australia	1.01	0.67, 1.54
Overweight x Canada	0.97	0.61, 1.53
Overweight x Mexico	1.15	0.81, 1.63
Overweight x USA	1.12	0.75, 1.66
Obesity x Australia	0.56	0.33, 0.93
Obesity x Canada	0.71	0.43, 1.16
Obesity x Mexico	0.93	0.60, 1.43
Obesity x USA	0.57	0.36, 0.91

[a] Interaction term = independent variable x country, reference country throughout = UK, interaction term added to separate maximally adjusted logistic regression models (Model 2).
